# Novel Therapeutic Targets in Cancers

**DOI:** 10.3390/ijms241914660

**Published:** 2023-09-28

**Authors:** Elena Levantini

**Affiliations:** Institute of Biomedical Technologies, National Research Council (CNR), Area della Ricerca di Pisa, 56124 Pisa, Italy; elena.levantini@itb.cnr.it

Cancer cells can arise in any organ of the body, and their cells of origin vary depending on the tissue type. In addition, any single tumor, independently of its location of origin, is not homogeneous itself, rather consisting of cells with diverse biological traits. To make matters worse, patients with the same diagnosis may have different molecular profiles of their tumor. Such heterogeneity poses a challenge for diagnosis, prognosis, and treatment of cancer, as it makes the tumor more adaptable, aggressive, and resistant.

In the last decades we have developed novel precision-oncology protocols that are starting to be adopted in routine clinical practice. However, despite major advances, the dream of converting solid tumors into a chronic disease is still unfulfilled, and long-term remission eludes us. Some of the current challenges in oncology deal with: (i) Identifying the molecular profile of each patient’s tumor and tailoring the therapeutic approach, rather than using a one-size-fits-all approach; (ii) Exploiting the tumor microenvironment (TME), the most tumor-intermingled organ that lives symbiotic to cancer cells [1], in order to enhance drug delivery and efficacy of treatment; (iii) Harnessing the power of immunotherapy to activate the body’s own defense system against cancer; (iv) Identifying new biomarkers and diagnostic tools to monitor treatment response and predict prognosis; (v) Combining different modalities of therapy to achieve synergistic outcomes, optimize benefits and minimize side effects.

This Special Issue (which was so well received that we also launched a 2.0 version) explored the latest advances in precision-oncology for solid tumors to tailor personalized and effective strategies for oncology patients by exploiting cancer cell vulnerabilities. This scientific anthology highlights the importance of understanding tumor heterogeneity, evolution, and recurrence, as well as developing novel therapeutic strategies. These strategies encompass addressing how RNA molecules, proteins, vesicles, and channels can modulate cancer progression and dissemination. They also cover how these components can be targeted or manipulated to bypass cancer cell’s growth and drug resistance by adopting different modalities, such as immunotherapy, metabolic inhibition, targeted therapy, epigenetic modification, and RNA regulation/modulation. The 15 papers in this collection offer a valuable insight into the molecular mechanisms and therapeutic potential of these emerging approaches in cancer treatment.

What if there was a way to target the most aggressive cancer cells that have the potential to spread to other organs? Suzuki et al. [2], developed a new monoclonal antibody (C_44_Mab-6) that can specifically detect CD44v3, a variant of CD44, a protein that facilitates tumor progression and dissemination by enhancing its stemness, invasiveness, and drug resistance. CD44v3 is expressed in various types of cancer, such as oral squamous and head and neck carcinomas, breast, pancreatic, and colorectal cancer. They discuss the potential of C_44_Mab-6 for novel therapeutic modalities, such as near-infrared photo-immunotherapy and chimeric antigen receptor (CAR) T-cell therapy, that could target CD44v3-expressing cancer cells more effectively. Another article by De Marco et al. [3] offers a comprehensive review on adoptive cell therapy, a promising approach that involves transferring genetically modified immune cells into patients to target and eliminate tumor cells. One of the most successful examples of adoptive cell therapy is the CAR T cell strategy. The authors review its challenges and opportunities, such as improving the safety, specificity, efficacy, and durability of the treatment. They also discuss the latest advances and future directions of CAR T cell approaches, such as using novel CAR designs, targeting multiple antigens, combining with other therapies, and expanding to solid tumors. This paper provides a valuable insight into the current state and potential of the CAR T cell method in cancer treatment. However, CAR-T cell therapy is not without limitations. Harrer et al. [4] discuss how several CAR products have received FDA and EMA approval for treating lymphoid leukemias and lymphomas. However, many patients are either refractory (due to a priori CAR-T-cell refractory disease) or experience relapse after CAR-T cell administration. This can be due to antigen shutdown, which means the tumor cells avoid detection by CAR-T cells, or CAR-T cell dysfunctionality, which means the CAR-T cells show insufficient proliferation and cytotoxicity. The authors review different approaches to combine CAR-T cells with small molecules or antibodies to boost their efficacy against both blood cancers and solid tumors. Farah et al. [5] report on the metabolic changes occurring in melanoma cells after they are challenged with the anti-PD1 antibody. This antibody blocks the PD-1 pathway, a sneaky mechanism that cancer cells use to escape from the surveillance of T cells. By blocking this pathway, the antibody unleashes the full power of T cells to fight against tumor cells. The authors found that such treatment not only slowed down tumor growth, but also reduced lactate production in cancer cells, indicating a shift in their metabolic state. This is relevant in that such metabolic shift may reduce: (i) their energy production and biosynthesis capacity, which could impair their proliferation and survival; (ii) their ability to acidify the TME, which could limit their invasion, metastasis, angiogenesis, and immune evasion; and (iii) their ability to provide lactate as a fuel or a signal for other cells in the TME, which could disrupt their metabolic symbiosis or slavery. They used a novel technique called in vivo metabolic spectroscopy to measure the levels of lactate, that can be adopted to monitor the early response to immunotherapy in melanoma patients, and tailor the treatment strategy based on the metabolic profile of each patient. The paper by González-Arriagada et al. [6] discusses the branch of immunotherapy that deals with chemokine receptor antagonist therapy. The authors suggest the potential use of HIV-associated chemokine receptor antagonists in cancer treatment. The CXCR4/CXCL12 and CCR5/CCL5 axes are exploited by HIV to enter and infect human cells. They have also been associated with the early (epithelial-mesenchymal transition and invasion) and late events (migration and metastasis) of tumor progression. Since drugs that block these receptors have been developed to treat HIV infection, the authors elaborate on how CCR5 and CXCR4 antagonists can also modulate the immune response against tumors. Although preclinical studies have shown promising results, clinical trials are needed to include these drugs in the oncological treatment protocols. The paper also discusses the prospects and pitfalls of integrating these drugs with other therapies, such as immunotherapy.

Another element that plays a role in tumor development and progression is quiescence, a state of dormancy in which cells stop dividing but remain viable. Quiescent cancer cells (QCCs) are non-dividing and resting cells that can escape most anti-cancer drugs and cause cancer recurrence. QCCs are prevalent and diverse in different types of solid tumors, and they can switch between quiescence and proliferation depending on the environmental cues, leading to cancer growth, recurrence, and metastasis. Lindell et al. [7] summarize the current knowledge on QCCs and acknowledge the challenges and limitations of QCC-targeted therapy, such as the lack of reliable QCC models and detection methods, the heterogeneity of QCCs, and the non-specificity of current interventions. They discuss the most updated strategies to target them, including: (i) Reactivating QCCs and removing them via cell-cycle-dependent anticancer agents; (ii) Modulating the quiescence-to-proliferation switch; (iii) Decreasing their stemness attributes; (iv) Exploiting their autophagic ability; and (v) Inhibiting their metabolism with specific drugs. The paper concludes that simultaneous co-targeting of proliferating and quiescent cancer cells may ultimately lead to the development of more effective therapeutic strategies for the treatment of solid tumors.

Another challenge for cancer treatment is acquired drug resistance (ADR), which occurs when tumor cells become resistant to anti-neoplastic drugs after exposure. Singh et al. [8] review the current knowledge on how STAT3, a signaling molecule that is often activated in malignant cells, contributes to the development of ADR to numerous anti-cancer therapies, such as chemotherapeutic agents, targeted kinase inhibitors, anti-hormonal drugs and monoclonal antibodies. STAT3 suppresses the immune system, interferes with autophagy and anoikis, and alters the function of p53 and NNMT, thus favoring tumor survival and growth. The authors suggests that using a STAT3 inhibitor along with other anti-cancer agents could prevent or reverse resistance and improve treatment outcomes by restoring normal cell death processes, correcting molecular dysfunctions, and enhancing immune system function.

A different approach to treat cancer is studying its epigenetics. Wu et al. [9] focused on histone acetyltransferase (HAT) inhibition strategies. In details, they report that the MOF/MSL (Males absent On the First/Male-Specific Lethal) HAT complex can acetylate the transcription factor Yin Yang 1 (YY1), which makes it more susceptible to ubiquitination and proteasomal degradation. Since YY1 plays an important role in the initiation and progression of various malignancies, and its expression levels are often correlated with cancer aggressiveness, metastasis, drug resistance, and poor prognosis, understanding the molecular mechanisms of the MOF/MSL HAT-YY1 axis may have important implications for cancer diagnosis and treatment. Eventually, modulating the interaction between the MOF/MSL HAT complex and YY1 could be a novel therapeutic tactic for tumors with aberrant YY1 activity.

Identifying partners in crime is important in cancer. De Rosa et al. [10] uncovered a deadly trio of proteins that fuel the growth of neuroblastoma, a rare and aggressive childhood malignancy. They found that the oncogene MYCN manipulates the cell cycle by disabling RB, the guardian of the genome, by hyperphosphorylation. RB normally inhibits E2F3 activity. By adding phosphate groups on RB, MYCN disables its function and eventually allows E2F3 to activate genes that promote cell cycle progression and DNA replication. MYCN and E2F3 are both prognostic markers of neuroblastoma, being associated with low survival rates in patients. The authors suggest that targeting the enzymes that phosphorylate RB or blocking the activity of MYCN and E2F3 could be a novel personalized-medicine plan against this devastating disease.

Cancer is a complex disease that involves the cooperation of various factors that promote its growth and survival. One such factor is their notorious ability to evade cell death. One of the mechanisms that tumor cells use to escape cell death is by altering their mitochondria, the organelles that produce energy and regulate cell survival. One such alteration can affect the mitochondrial permeability transition pore (mPTP), a channel located in the inner mitochondrial membrane. The opening of the mPTP can trigger apoptosis, or programmed cell death, by releasing cytochrome c and other pro-apoptotic factors from the mitochondria into the cytosol, where they activate the caspase cascade. Normal cells open the mPTP in response to high mitochondrial Ca^2+^, oxidative stress, and depolarization. However, cancer cells prevent or limit the mPTP opening and avoid apoptosis by altering their mitochondrial structure and function. How can we target the mPTP to induce cell death in tumor cells? This is the question that Waseem et al. [11] address in their review article, where they discuss the role of the mPTP in cancer and its potential as a novel anti-cancer target. They also present the latest advances and future directions of mPTP-targeting drugs/molecules that can treat cancer.

Malignant cells can communicate with other cells through extracellular vesicles (EVs), which are small membrane-bound particles that can carry various cargo, such as proteins, nucleic acids, lipids, and metabolites, from the originating cell to a recipient cell. EVs can modulate many biological processes, such as cell communication, immune response, and tissue repair. However, EVs can also contribute to cancer development and progression by promoting drug resistance; consequently, they can facilitate tumor growth and spread. The paper by Yi et al. [12] provides an informative synopsis of the oncogenic signaling mediated by EVs derived from cancer cells and their potential as a novel target for anti-cancer therapy. In this review, they also point out the obstacles of this approach: the heterogeneity of EVs, the lack of specific markers, and the difficulty of isolating and characterizing them. Using triple-negative breast cancer cells as an example, they suggest ways to prevent EVs from being pro-tumorigenic, such as by blocking their uptake, secretion, or cargo, and enhancing the immune response against cancer-EVs.

Another emerging approach in tumor intervention is to use small molecule therapy, a type of targeted therapy that uses compounds that can enter the cells and interfere with their function or survival. Sawicka et al. [13] investigated the anticancer potential of a new class of small molecules, called lithocholic acid-based imidazolium salts (LCA-IMSs), against colon cancer cells. They synthesized different LCA-IMSs with varying carbon chain lengths, and evaluated their cytotoxicity in vitro and in vivo. They found that the length of the carbon chain influenced the efficacy of the LCA-IMSs, and that one of them (S6) had a superior antitumor activity compared to the conventional chemotherapeutic agent 5-fluorouracil (5-FU) in a mouse xenograft model: S6 induced apoptosis and cell cycle arrest in colon cancer cells, and significantly reduced tumor volume and weight in mice. Therefore, this study suggests that LCA-IMSs are a promising compounds for the treatment of colon cancer, and that S6 is a potential candidate for further development and clinical trials.

RNA molecules are versatile and dynamic regulators of gene expression and cellular functions. By binding to other RNA molecules or proteins, they can modulate RNA fate and function. Abnormal interactions can lead to tumor development and progression, by affecting various aspects of cancer biology, such as metabolism, proliferation, invasion, angiogenesis, and drug resistance. Therefore, one of the emerging strategies for cancer treatment is RNA therapy. In this Special Issue, several research articles explore the role and potential of RNA-based intervention in different types of tumors, using various approaches and techniques.

One of the RNA-binding proteins (RBPs) that has attracted considerable attention in recent years, Insulin-like growth factor 2 mRNA-binding protein 3 (IGF2BP3), is a component of the m6A reader complex, which recognizes the m6A modification on RNAs. m6A methylation is the most common and reversible RNA modification in eukaryotes, and it affects various aspects of RNA biology such as: stability, splicing, translation, and localization. Liu et al. [14] compiled a detailed summary of the physiological and pathological roles of IGF2BP3 in organisms and tumors. The authors of this article highlight the potential clinical applications of IGF2BP3 as a biomarker for diagnosis, prognosis, and prediction of response to therapy in cancer patients. They also suggest that targeting IGF2BP3 could be a promising therapeutic approach for cancer treatment. However, RNA-binding proteins are not the only molecules that can influence cancer development and progression. Another factor that can modulate gene expression and cellular functions in tumor cells are microRNAs (miRNAs). These molecules are small non-coding RNAs that can regulate gene expression by binding to messenger RNA and preventing its translation into protein. They influence various cellular functions via their dynamic and complex interactions, including cancer initiation, progression, and metastasis. Therefore, another emerging method for oncological treatments is microRNA therapy, which aims to modulate the expression and function of microRNAs in tumor cells. The microRNA strategy has two main approaches: miRNA replacement and miRNA inhibition. Two examples of the first kind are described in this Special Issue. Casanova et al. [15] studied miR-223 in rhabdomyosarcoma (RMS), a type of muscle cancer that affects adolescents and young adults (AYA) more severely than younger children. They found that miR-223 expression was lower in RMS than normal tissues. They also showed that restoring miR-223 in RMS cells reduced their growth and invasion, probably by targeting IGF1R, a protein that controls cell survival and is involved in AYA-RMS. MiR-223 is also made by the large numbers of immune cells in AYA-RMS tumors, which means it may have different roles and effects depending on the cell type and the context. For example, it may suppress tumor growth by targeting IGF1R in cancer cells, but it may also help tumor escape by modulating the immune response in immune cells. Therefore, it is important to understand how miR-223 affects the interaction between tumor and immune cells in AYA-RMS, as the TME is a complex system that influences the disease outcome. Tang et al. [16] investigated miR-503 in head and neck cancer (HNC). They showed that miR-503 is a tumor suppressor that can stop cell invasion by lowering the expression of WNT3A and its related molecules, such as enzymes that can break down the matrix and help cell invasion. The Wnt pathway is important for cell development and survival, but it can also be hijacked by tumor cells to promote their growth and invasion. This paper suggests that enhancing miR-503 may be a new way to stop cancer invasion.

In conclusion, the novel therapies described in this Special Issue have shown promising results for treating a variety of different solid tumors. However, there are still many challenges and obstacles to overcome before these therapies can be widely applied in clinical practice. Future research should focus on improving the safety, specificity, and durability of these therapies; in addition to exploring the synergies among these novel agents, as well as their combination with treatments that are already available.
